# Policy implications of Codex Alimentarius guidelines on nutrition labelling

**DOI:** 10.2471/BLT.24.292695

**Published:** 2025-07-09

**Authors:** Katherine Shats, Kyra Berasi, Anne-Marie Thow, Alexandra Jones

**Affiliations:** a United Nations Children’s Fund, 3 United Nations Plaza, New York, NY 10017, United States of America.; b University of Sydney, Sydney, Australia.; c The George Institute for Global Health, Sydney, Australia.

Unhealthy diets are a leading cause of death and disability globally, largely driven by the widespread availability of ultra-processed, pre-packaged foods. To combat this situation, the World Health Organization (WHO) has endorsed front-of-pack nutrition labelling as a good policy to aid consumers to make healthier choices.^1^ As of mid-2025, at least 40 countries, of varying income levels and geographic locations, had adopted various front-of-pack nutrition labelling systems, including mandatory, nutrient-specific warning labels and overall scoring systems, which are gaining traction.^2^

Governments that have introduced evidence-based front-of-pack nutrition labelling measures have faced considerable resistance from the food and beverage industry and their allies, including through legal and trade threats.^3^ Industry has particularly opposed mandatory and interpretive front-of-pack nutrition labelling systems (using symbols, graphics and colours), which are now supported by a growing body of evidence that clearly shows these are more effective than voluntary, non-interpretive systems, which tend to produce poorer outcomes for consumers.^4^ Almost all proposals for mandatory, interpretive labelling measures have had specific trade concerns raised about them at the World Trade Organization (WTO) Technical Barriers to Trade Committee.^3^ These specific trade concerns often questioned whether proposed labelling initiatives were consistent with relevant international standards: in this case, provisions on labelling developed by the international food standards agency, the Codex Alimentarius Commission (Codex). Although none of these concerns has so far escalated to more formal dispute, responding requires substantial governmental resources and may deter governments from pursuing effective policy measures. Against this backdrop, the continued rise in front-of-pack nutrition labelling adoption and concerns about trade impacts prompted Codex to negotiate new international guidelines on this labelling between 2017 and 2021.

In this article, we clarify the role of the guidelines and how they can be used to advance national efforts to address unhealthy food environments. 

## Opportunities and risks at Codex

As with much of Codex’s work, there was greater participation from powerful food industry representatives than public health and consumer voices in the early stages of the guideline development process.^5^ For example, in Codex’s first electronic working group on front-of-pack nutrition labelling, there were 40 country participants and 20 observers, including 14 nongovernmental organizations representing the food industry and only four representing consumer and public health interests.^5^ This disparity in numbers raised concerns that industry influence could result in guidelines that would curtail the ability of governments to design fit-for-context, evidence-based front-of-pack labelling that would have the desired public health impact. For example, by only allowing for industry-developed or weaker and less evidence-based labelling systems, by restricting the use of mandatory front-of-pack nutrition labelling, or by limiting the ability of governments to adapt and evolve their labelling systems as new evidence emerges.

On the other hand, the public health community welcomed the opportunity for new Codex guidelines that could recognize the growing evidence base for a range of effective front-of-pack nutrition labelling systems and provide governments a basis to continue to develop legitimate policy measures designed to protect the health of their populations. To this end, public health groups articulated the need for a text that allowed flexibility for governments to adopt evidence-based front-of-pack nutrition labelling tailored to the needs of their own populations, that recognized the need for government leadership and strong conflict of interest safeguards, and could be based on general principles rather than rigid rules, which could limit policy innovation.^5^

## The final guidelines

After five years of negotiations, Codex guidelines on front-of-pack nutrition labelling were adopted as Annex 2 to the existing Codex Guidelines on Nutrition Labelling in 2021.^6^ Increased engagement and coordination among public health and consumer advocates throughout negotiations enabled their contributions to shape the outcome of the final text.

While the final guidelines do not specify a single best-practice approach or fully align with more detailed WHO normative guidance on front-of-pack nutrition labelling,^7^ they nevertheless represent an important victory for public health in holding industry influence at Codex at bay. This success is partly seen in what the guidelines do not prescribe, as they remain broad enough to allow countries the flexibility to pursue innovative policies. In particular, the guidelines allow Member States to retain flexibility to select the most appropriate front-of-pack nutrition labelling system for their context, with explicit recognition of the legitimacy of mandatory measures. The guidelines also require the format of front-of-pack nutrition labelling to be supported by scientifically valid consumer research, thus supporting a shift away from industry-preferred systems that are not evidence-based. Finally, the guidelines also provide that the development of front-of-pack nutrition labelling should be government-led but developed in consultation with other interested parties, a pronounced shift from earlier drafts prescribing a need for collaboration with stakeholders including industry.

Table 1 provides a summary of the guideline provisions with contextual information from the final round of negotiations.

**Table 1:** Text of the Codex Guidelines on Front-of-Pack Nutrition Labelling and contextual information about the final round of negotiations by the Codex Committee on Food Labelling in its forty-sixth session (27 September to 1 October 2021)

Section and text^a^	Background information from Report of Codex Committee on Food Labelling forty-sixth session^b^
**1. Purpose**	
Provide general guidance to assist in the development of front-of-pack nutrition labelling, a form of supplementary nutrition information, as a tool to facilitate the consumer’s understanding of the nutritional value of the food and their choice of food, consistent with the national dietary guidance or health and nutrition policy of the country or region of implementation.	The development of the guidelines’ purpose involved discussions about whether they should be prescriptive or flexible. During the meeting, the Chair of the working group reminded the committee that the guidelines are designed to be flexible so they cover the range of front-of-pack nutrition labelling systems currently in place and leave room for new ones to be established in the future.
**2. Scope**	
2.1 These Guidelines apply to front-of-pack nutrition labelling (FOPNL) to be used on pre-packaged foods. FOPNL should only be provided in addition to, and not in place of, the nutrient declaration subject to Section 5 of the *Guidelines on Nutrition Labelling* (CXG 2–1985). 2.2 Foods covered by the following Codex standards are excluded: *Standard for Infant Formula and Formulas for Special Medical Purposes Intended for Infants* (CXS 72–1981) *Standard for Follow-up formula* (CXS 156–1987) *Standard for Labelling of and Claims for Foods for Special Medical Purposes* (CXS 180–1991). In addition, other foods could be considered for exclusion at a national level dependent on the type of FOPNL being developed, such as alcoholic beverages and other foods for special dietary uses. FOPNL should not be used in any way that could promote the consumption of alcohol. 2.3 Certain pre-packaged foods may be exempted from FOPNL. Exemptions from FOPNL should align with the exemption from the nutrient declaration as described in Section 3.1.2 of the *Guidelines on Nutrition Labelling* (CXG 2–1985). 2.4 These Guidelines can also be used as a guide in the case where simplified nutrition information is displayed near the food (e.g. shelf-tags or food service), for unpackaged foods or for foods sold via online (e.g. information available at point of purchase on websites).	The meeting report notes the committee’s overall support for the proposed scope. Concerns were raised during the meeting by one Member State regarding whether the list of exclusions should be broader. The committee concluded that the proposal would remain unchanged, noting that it “had received strong support in the virtual working group and allowed for additional exclusion decisions to be taken at the national level”. The committee further explained that “[t]his was consistent with the approach to keep the guidelines high level and flexible to support all front-of-pack nutrition labelling systems”.
**3. Definition of front-of-pack nutrition labelling**	
For the purposes of these Guidelines: 3.1 *Front-of-pack nutrition labelling (FOPNL)* is a form of supplementary nutrition information that presents simplified nutrition information on the front-of-pack of pre-packaged foods. It can include symbols/graphics, text or a combination thereof that provide information on the overall nutritional value of the food and/or on nutrients included in the FOPNL. 3.2 FOPNL can be voluntary or mandatory in line with national legislation.	The electronic working group Chair noted at the committee meeting that the question of whether the guidelines should explicitly state that front-of-pack nutrition labelling could be voluntary or mandatory remained unanswered. The Chair proposed that committee consider including the statement “FOPNL can be voluntary or mandatory” in the definition. After deliberation, the committee agreed to amend the definition by including the statement: “FOPNL can be voluntary or mandatory in line with national legislation”. This decision was guided by the goal of keeping the guidelines flexible and accounting for the range of front-of-pack nutrition labelling systems that currently exist and may be developed in the future. This decision was also informed by the Codex Secretariat’s clarification around the meaning of “should” in section 5 of CXG 2–1985. Settling a long-standing question, the Secretariat clarified that the word “should” had “provided flexibility for front-of-pack nutrition labelling to be either voluntary or mandatory”. The Chair explained that the decision regarding whether any particular front-of-pack nutrition labelling scheme is voluntary or mandatory is to be made by competent authorities.
**4. Principles for the establishment of front-of-package nutrition labelling systems**	
In addition to the general principles in the General Standard for the Labelling of Prepackaged Foods (CXS 1- 1985), a FOPNL should be based on the following principles: Only one FOPNL system should be recommended by government in each country. However, if multiple FOPNL systems coexist, these should be complementary, not contradictory to each other. FOPNL should be applied to the food in a manner consistent with the corresponding nutrient declaration for that food. FOPNL should align with evidence-based national or regional dietary guidance or, in its absence, health and nutrition policies. Consideration should be given to the nutrients and/or the food groups which are discouraged and/or encouraged by these documents. FOPNL should present information in a way that is easy to understand and use by consumers in the country or region of implementation. The format of the FOPNL should be supported by scientifically valid consumer research. FOPNL should be clearly visible on the package/packaging at the point of purchase under normal conditions. FOPNL should help consumers to make appropriate comparisons between foods. FOPNL should be government led but developed in consultation with all interested parties including private sector, consumers, academia, public health associations among others. FOPNL should be implemented in a way that facilitates the broad availability of FOPNL for consumer use. FOPNL should be accompanied by a consumer education/ information program to increase consumer understanding and use of FOPNL in line with government recommendations. FOPNL should be monitored and evaluated to determine effectiveness and impact.	The committee considered numerous proposed edits to the principles during its meeting. One important issue was whether and how to include conflict-of-interest safeguards in the guidelines. Some observers recommended that the guidelines explicitly mention conflict-of-interest safeguards. The committee decided to use a less direct approach, and intentionally – although implicitly – included language related to safeguarding against conflicts of interest in the principle stating, “Front-of-pack nutrition labelling should be government led but developed in consultation with all interested parties including private sector, consumers, academia, public health associations among others”. The Chair explained that two key amendments to the text of this principle were meant “to recognize that governments could implement conflict-of-interest safeguards.” First, it states that front-of-pack nutrition labelling development should be “government led.” Second, the word “consultation” replaced the word “collaboration,” which reinforces governments’ leadership in developing front-of-pack nutrition labelling and ability to implement safeguards against conflicts of interest. Another extensively discussed topic was whether to add a principle that “Front-of-pack nutrition labelling should be objective and non-discriminatory,” which was proposed by the European Union and its Member States in the virtual working group. The committee rejected this proposal after many points were made opposing it, including: According to the working group Chair, “the term ‘non-discriminatory’ was neither used nor defined” in other Codex texts, and “could lead to misinterpretation and confusion”; The principle of non-discrimination “was inherent in the trade obligations that are already in Codex texts”; “Front-of-pack nutrition labelling was evidence-based and uses nutrient profiles as an objective measure to discriminate between foods”; Not exploiting fear in consumers is a concept that “was already covered by other Codex guidelines”; The guidelines’ flexibility at the national or regional level “allows for differences for foods that might be recommended as part of a healthy diet in a different country or region”; “Front-of-pack nutrition labelling should not discriminate foods but provide consumers with supplementary information to facilitate their choices as defined in the purpose”.

^a^ The text is verbatim from Codex Guidelines on Front-of-Pack Nutrition Labelling.^6^

^b^ Adapted from the Codex Committee on Food Labelling.^8^

The guidelines provide much-needed support to Member States seeking to introduce front-of-pack nutrition labelling that is evidence-based, in line with emerging global best practice and free from commercial conflicts of interest. Legal and trade threats might continue as a persistent industry strategy to delay progressive public health action. However, it is important for Member States and the public health community to appreciate that if front-of-pack nutrition labelling policies are developed according to the high-level principles set out in the new Codex guidelines, these kinds of arguments raised against such measures in trade committees no longer have merit.

## The current challenge

Despite these important outcomes, the guidelines are not yet used effectively in key trade committees. WTO Member States continue to raise arguments similar to those made before their adoption, including queries about consistency of national actions with international standards and concerns over a lack of international harmonization, although the guidelines now provide a clear basis of defence against such allegations.^9^^,^^10^

Most importantly, governments should be encouraged to reference the guidelines when notifying their proposed front-of-pack nutrition labelling measures to relevant trade forums. Additionally, governments should reference the guidelines when responding to concerns raised by other Member States to confirm that their measures are based on relevant international standards.

The guidelines should also be used to guide government stakeholder engagement plans for front-of-pack nutrition labelling policy development. The prominence of discussions on conflicts of interest throughout the guideline development process underscored growing concerns about protecting government decision-making from industry interference. The guidelines unequivocally state that the development of front-of-pack nutrition labelling should be government-led,^6^ affirming governments as leaders for labelling and managers of potential conflicts of interest held by food industry stakeholders who profit from the sale of unhealthy foods. This approach aligns with WHO’s normative guidance on engaging with the private sector in nutrition policy-making,^11^ similar to the broad guidance on limiting industry interference in tobacco policy-making contained in Article 5.3 of the Framework Convention on Tobacco Control.^12^

A key strength of the guidelines is that they support governments to respond to a rapidly evolving evidence base, reflecting the need for ongoing innovation in national and regional policies. For example, this inherent flexibility allows governments wishing to respond to the growing evidence about the health harms of ultra-processed foods to incorporate these considerations into both nutrient profiling and front-of-pack nutrition labelling systems. Similarly, the guidelines provide support to countries with existing such labelling measures to review and revise these measures in accordance with new evidence.

The principles-based approach of the guidelines leaves space for and necessitates ongoing WHO normative guidance to clarify best practices, support continued policy innovation and respond to new challenges. Sustained political commitment from Member States and advocates will also be critical to counter persistent industry opposition and ensure effective implementation.

The Codex guidelines on front-of-pack nutrition labelling provide a flexible and clear framework for national policy-makers to develop evidence-based, government-led such labelling systems that effectively address public health needs. Yet the guidelines’ potential positive impact on national front-of-pack nutrition labelling policy development will only be realized if governments know and understand them and then put into practice. By empowering policy-makers to adapt the latest scientific evidence to national or regional contexts, these guidelines support innovative measures to promote healthier diets and combat diet-related noncommunicable diseases.
